# Mothers' perceived proximity to green space is associated with TV viewing time in children: The Growing Up in Scotland study

**DOI:** 10.1016/j.ypmed.2014.11.018

**Published:** 2015-01

**Authors:** Daniel Aggio, Lee Smith, Abi Fisher, Mark Hamer

**Affiliations:** a Health Behaviour Research Centre, University College London, London, UK; b Physical Activity Research Group, Department of Epidemiology and Public Health, University College London, UK

**Keywords:** Sedentary behaviour, Physical activity, Green exercise, Environment

## Abstract

**Objective:**

The aim of this study is to investigate whether mothers' perception of distance from home to green/open spaces is associated with their child's screen time.

**Method:**

We used mother-reported data from sweep six (2010–2011) of the Growing Up in Scotland study (*n* = 3586 children aged 5.9 yrs) to examine associations between walking distance from home to green/open space and screen time (TV viewing time/computer use). Analyses were adjusted for age, sex and other pre-specified covariates, including sport/exercise participation, mental and general health, birth weight, parental socio-economic group (SEG) and smoking status.

**Results:**

Children living the furthest distance from green/open spaces (> 20 minutes' walking distance) displayed over 2 h (95% CI, 0.65 to 3.51) more weekly TV time than the reference category (< 5 minutes' walking distance). Compared to children in the reference category, those in the > 20 minute category had worse mental health (mean SDQ [Strengths and Difficulties Questionnaire] score ± SD, 7.0 ± 4.6 vs. 8.7 ± 6.2) and general health (% fair–poor, 4.6 vs. 8.6), and were more likely to come from lower SEG households.

**Conclusion:**

Mothers' perceived distance from home to green/open spaces was associated with child's TV time at age 5.9 years.

## Highlights

• Children (aged 6 years) with mothers perceiving to live further from green/open spaces watch more TV.
• They also have worse mental and general health.
• Mothers' perceived distance from home to green/open spaces was not associated with child computer usage.

## Introduction

Sedentary behaviour (e.g. sitting) is associated with several cardiometabolic risk factors independent of physical activity in young people (Vaisto et al., 2014). Moreover, it is associated with lower self-esteem and academic performance (Tremblay et al., 2011). Screen time contributes to approximately half of total sedentary time in young people aged 9 to 16 years (Klitsie et al., 2013; Olds et al., 2010). Over half of boys and a third of girls (aged 10 years) engage in more than the recommended maximum 2 h a day of screen time (Klitsie et al., 2013). By adolescence, more than 70% exceed this recommendation (Owens et al., 2013).

The association between accessible green/open spaces, such as parks and recreational spaces, and health outcomes is well established (Takano et al., 2002; Maas et al., 2006; Beyer et al., 2014). There is also evidence that exercising in green environments is more beneficial to psychological wellbeing than exercising indoors (Thompson Coon et al., 2011). Children (aged 10–12 years) are inherently more active when outside (Cooper et al., 2010; Cleland et al., 2008) and some research suggests that access to green/open spaces is associated with higher physical activity levels (Mytton et al., 2012; Epstein et al., 2006), but this is opposed by other evidence demonstrating no association (Maas et al., 2008; Foster et al., 2009). This conflicting evidence may be due to a lack of continuity in how access to green/open spaces was measured. Some studies used an area of 3–4 km^2^ (Mitchell and Popham, 2008; Maas et al., 2008) to assess the amount of green space within that location and others used a larger area (Mytton et al., 2012). There is also an inconsistency in how physical activity was measured, some using self-report (Foster et al., 2009; Maas et al., 2008; Mytton et al., 2012) and others using objective measures (Epstein et al., 2006). Parental perceptions about the physical environment are, however, consistently associated with child's physical activity. One explanation may be that of independent mobility (Fyhri and Hjorthol, 2009), whereby children whose parent's perceive the environment as ‘safe’, and thus give their child greater independence, are more likely to be active. On the other hand parents who perceive the environment to be “unsafe” may restrict children's play to the home environment. Features of the home environment, such as the number of household TVs, are known to be associated with sedentary behaviours (Kaushal and Rhodes, 2014) so restricting children's play to the home environment may promote engagement in sedentary activities.

Physical environmental features, such as proximity to green/open spaces, are associated with sedentary behaviours in adults (Storgaard et al., 2013) but evidence for this association in young people is lacking. The limited existing evidence suggests that parental perception of a safe neighbourhood is associated with reduced TV time in children aged 2–3 years (Burdette and Whitaker, 2005). Conversely, a study in children aged 2 years found that mothers' perception of a safe outdoor play environment (i.e. having good parks/playgrounds/play spaces in the neighbourhood) was not associated with TV time (Xu et al., 2014). Neither of these studies, however, investigated the impact of distance from home to green/open spaces on screen time, which is reportedly a factor associated with physical activity levels (Mytton et al., 2012; Epstein et al., 2006). Evidently, parents are influential in the development of lifestyle behaviours in young people, but how parental perceptions of the environment affect young people's engagement in sedentary pursuits is not well understood. Thus, this study aimed to investigate whether mothers' perception of distance from home to green/open spaces is associated with their child's screen time.

## Methods

### Study design

The Growing Up in Scotland (GUS) study is a longitudinal social survey which follows the lives of Scottish children from infancy through to their teens, and aims to provide important new information on young children and their families in Scotland (http://growingupinscotland.org.uk). In 2005 a birth cohort of 5217 participants was established (63% response rate). Families were selected at random from Child Benefit records. The present analyses incorporated data from sweep six (*n* = 3657 participants; 70% of original cohort) when children were, on average, aged 5.9 yrs. Mothers or carers provided informed consent and a parental interview sought information on the child's home background, social experience, and a number of factors concerning the experiences of the child and the family. The information was gathered through a face-to-face computer-assisted-personal-interview in participants' homes. In accordance with the University College London Research Ethics Committee Guidance, ethical approval was not required for analyses of anonymous secondary data although GUS has received full ethical approval from the Scotland Multi-Centre Research Ethics Committee.

### Sedentary behaviour variables

Information was recorded on TV viewing frequency and duration. “Thinking about the past week, on how many days did your child watch television, including watching DVDs or videos, for at least 10 min at a time?” “How long would your child usually watch television for in total on an average day?” These data were used to derive total weekly TV viewing time (hours per week). Similar questions were asked regarding the child's use of computers, laptops or games consoles (eg, Nintendo DS or Wii, Sony PSP, Playstation, Xbox) in order to derive total weekly computer time (hours per week). While we acknowledge that these specific questions have not been validated, other proxy report tools for determining child sedentary behaviour have shown to be valid (Wen et al., 2010) and reliable (Salmon et al., 2006).

### Main exposure and covariates

Mothers were asked questions about green or open spaces in their local area and were specifically asked “how far away from your home is the nearest green or open space?” Response options were: < 5 min walking distance; 5–10 min; 11–20 min; 21–30 min; > 30 min. The two most extreme categories were collapsed prior to analyses. Information was collected on the child's participation in sport [“In the last week, has your child done any sports or exercise activities (not counting things done as part of school lessons)?”] and were prompted with a card to show examples of activities their child might have taken part in. In addition, mothers recorded their child's frequency of outdoor play (days per week) and how frequently they took their child to the park or playground (daily; once or twice a week; less than weekly). Psychological distress was assessed using the parental version of the Strengths and Difficulties questionnaire (SDQ), which has demonstrated good reliability and validity (Goodman, 2001). A total score (ranging from 0 to 40) was calculated by adding the scores from the SDQ subscales of hyperactivity, emotional symptoms, conduct problems, and peer problems, with a higher score representing worse mental health. Mothers also provided a rating of their child's overall health, categorised as: excellent; very good; good; fair; poor.

Mothers provided information on their occupation, which was categorised using the National Statistics Socio-economic Classification [N-SEC] (Managerial/Professional/Intermediate [skilled & non-skilled]/Routine and manual), and also provided information about their smoking habit (none/current). Mothers reported their child's birth weight.

### Statistical analysis

We examined associations between living distance from green/open space and sedentary behaviours (weekly TV viewing time and computer use) using general linear models. The exposure (distance to green space) was analysed as a categorical variable from which coefficients were generated. The *p*-trend value was generated by entering the exposure as a continuous variable. TV viewing and computer use were treated as linear dependent variables. Initially we performed a basic analysis adjusting for age and sex. We then adjusted the models for the following covariates: child's sports participation, child's general health, SDQ score, birth weight, parental N-SEC and parental smoking habit. Pre specified covariates were chosen because they were hypothesised to be associated with both the exposure and outcome in the main analyses (Hamer et al., 2009). All data were weighted for analyses using a variable derived from SES, age, employment status, income and whether respondents were lone-parents, to ensure that Sweep 6 respondents were representative of population estimates. All analyses were conducted using SPSS version 22.

## Results

A total of 3586 children had complete mother-reported data and were included in the analysis. Descriptive statistics, categorised by walking distance from green/open space, including measures of TV viewing, engagement in sport/exercise and other covariates are shown in Table 1. TV viewing was significantly higher in the > 20 min category compared to the < 5 min category. Those in the > 20 min category also had significantly higher SDQ scores, were taken to parks/playgrounds by parents less frequently, had a worse general health rating, and were more likely to come from lower SEG households compared to those in the < 5 min category. Overall, 32.1% of the sample spent more than the recommended maximum for TV viewing of 2 h/day.

Table 2 presents the association between walking distance from home to green space and TV viewing. There was a linear association between distance from green space and TV viewing that was minimally attenuated after adjustment for all covariates; children in the > 20 min category displayed over 2 h (95% CI, 0.65 to 3.51) more weekly TV time than the reference category in the final adjusted model. The same models both showed no association between walking distance to green space and computer usage as shown in the Table 3.

## Discussion

To date, evidence for the association between the environment and screen time is inconsistent (Burdette and Whitaker, 2005; Xu et al., 2014). The results of this study, however, suggest that mothers' perceived walking distance from home to green/open spaces is associated with their child's weekly TV viewing time. Children living > 20 minutes' walk away from home to green/open spaces watched an extra 2 h of TV per week compared to those living < 5 minutes' walk away. There was, however, little evidence to suggest that distance from green/open spaces has any association with child computer usage, potentially because computer usage in this age group is less common (15.2% of the sample did not report any use of computers compared to 5.5% that did not view any TV).

In accordance with this study, similar papers have shown that neighbourhood environmental factors are associated with TV time in young people (Burdette and Whitaker, 2005) and adults (Sugiyama et al., 2007). However, to our knowledge, this is the first paper to provide evidence for the associations between distance to green space and TV viewing. Furthermore, while previous research has suggested that an increase in physical activity may explain the association between health and access to green/open spaces (Mitchell and Popham, 2008); here the results show that a reduction in TV time may also be a factor. A possible explanation for the association between mothers' perceived walking distance from home to green/open spaces and their child's weekly TV viewing time may be that children living in close proximity to green/open spaces have increased independent mobility, engaging in more physical activity within these environments and thus spending less time in sedentary pursuits, such as TV viewing. Nevertheless, this was not supported in the present study as there were no differences in the frequency of outdoor play in relation to living distance from green space. Another possible explanation is that parents are more willing to take their children to the park or playground as parents living < 5 min from green space were more likely to make daily visits to the park compared to those living > 20 min away (11.5% vs. 6.7%, *p* = 0.001). However, further adjustment for frequency of parent supervised park visits did not alter the association between green space and TV viewing (data not shown); thus other unmeasured factors (such as travel mode, social and economic resources) are likely to be involved. Sedentary time is independently associated with cardiometabolic risk factors (Vaisto et al., 2014) and approximately a third of the present sample exceeded the maximum time recommended to engage with entertainment media. This suggests that interventions to reduce sedentary time are warranted.

Interestingly, psychological distress was higher in participants reporting the furthest distance from home to green/open spaces compared to the shortest, supporting the notion that greater access to green/open spaces may promote psychological health, due to reduced stress, anxiety and depression (Beyer et al., 2014).

The GUS study is a large nationally representative sample of the Scottish population, which is a key strength of these analyses. The primary limitation of this study is the cross-sectional design, meaning the direction of causality for the association between mothers' perceived distance to green/open spaces and child's TV viewing time cannot be determined. A proxy report measure of total weekly TV time was used and therefore dependence on mothers' recall was a limitation. Although parental proxy-report measures tend to have acceptable validity (Lubans et al., 2011), factors such as social desirability bias may lead to an underestimation of TV-time. If measurement error was randomly distributed across exposure categories, it is unlikely to have influenced the findings. Nevertheless non-random biases could have weakened the associations found.

## Conclusion

In this sample, mothers' perceived distance from home to green/open spaces was associated with their child's TV time at 5.9 years of age.

## Conflict of interest

None of the authors have any competing interests to declare.

## Figures and Tables

**Table 1:** Descriptive characteristics of the Growing up in Scotland 2010–2011 sweep six cohort (*n* = 3586).

Variable		Walking distance from green/open space			
	Overall sample	< 5 min (*n* = 2446)	5–10 min (*n* = 819)	11–20 min (*n* = 217)	> 20 min (*n* = 105)
Child age (months)	70.2 ± 0.5	70.3 ± 0.5	70.2 ± 0.5	70.2 ± 0.4	70.2 ± 0.5
Child sex (% boys)	50.9	52.1	48.4	49.3	49.5
Birth weight (g)	3412.1 ± 594.0	3424.8 ± 595.5	3385.7 ± 578.3	3384.6 ± 614.9	3371.1 ± 636.6
Parental SEG (% professional occupation)	41.5	42.4	41.4	36.9	31.4
Parent smoker (%)	22.4	22.5	23.1	19.8	20.0
Child participation in any sport/exercise last week (%)	71.5	72.4	69.2	71.9	66.7
Frequency of outdoor play (occasions per week)	5.0 ± 2.5	5.1 ± 2.1	4.8 ± 2.5	4.7 ± 2.6	5.0 ± 2.4
Frequency child taken to park/playground (% daily)	10.4	11.5	9.2	5.1	6.7
Child TV viewing (h/wk)	9.4 ± 7.5	9.2 ± 7.1	9.6 ± 7.9	10.5 ± 7.0	11.7 ± 11.9
Child SDQ score	7.1 ± 4.8	7.0 ± 4.6	7.3 ± 4.9	7.6 ± 5.1	8.7 ± 6.2
Child general health (% fair–poor)	4.9	4.6	4.8	7.8	8.6

Data presented as mean ± SD or percentage where denoted. SEG, socio-economic group; SDQ, Strengths and Difficulties Questionnaire.

**Table 2:** Association between self-reported living distance from green space and weekly TV viewing hours in the Growing up in Scotland 2010–2011 sweep six cohort.

Walking distance from green space	Model 1 B coefficient (95% CI)	Model 2 B coefficient (95% CI)
< 5 min	Reference	Reference
5–10 min	0.38 (− 0.21, 0.97)	0.25 (− 0.33, 0.83)
11–20 min	1.31 (0.27, 2.34)	1.16 (0.14, 2.17)
> 20 min	2.49 (1.03, 3.95)	2.09 (0.65, 3.51)
*p*-trend	< 0.001	0.001

*Model 1* adjusted for age and sex of child. *Model 2* additionally adjusted for birth weight, parental SEG, parental smoking habit, child's participation in sport/exercise, child's Strength and Difficulties score, and child's general health.

**Table 3:** Association between self-reported living distance from green space and average daily computer use in the Growing up in Scotland 2010–2011 sweep six cohort.

Walking distance from green space	Model 1 B coefficient (95% CI)	Model 2 B coefficient (95% CI)
< 5 min	Reference	Reference
5–10 min	0.06 (0.002, 0.13)	0.05 (− 0.01, 0.11)
11–20 min	0.03 (− 0.08, 0.15)	0.03 (− 0.08, 0.14)
> 20 min	0.10 (− 0.05, 0.25)	0.08 (− 0.07, 0.23)
*p*-trend	0.04	0.10

*Model 1* adjusted for age and sex of child. *Model 2* additionally adjusted for birth weight, parental SEG, parental smoking habit, child's participation in sport/exercise, child's Strength and Difficulties score, and child's general health.
